# Biochemical analyses of a new GH18 chitinase from *Beauveria bassiana* KW1 and its synergy with a commercial protease on silkworm exuviae hydrolysis

**DOI:** 10.1128/aem.01285-25

**Published:** 2025-09-15

**Authors:** Yizhou Liu, Wenke Xiong, Xinhai Wang, Shuangcheng Liang, Lixin He, Xiaoqin Lin, Sidi Wang, Ying Zhang, Ruoting Zhan, Kui Wang

**Affiliations:** 1 Research Center of Chinese Herbal Resource Science and Engineering, School of Pharmaceutical Sciences, Guangzhou University of Chinese Medicine, Guangzhou, Guangdong, China; 2 Key Laboratory of Chinese Medicinal Resource from Lingnan (Guangzhou University of Chinese Medicine), Ministry of Education of the People’s Republic of China, Guangzhou, China; 3 Department of General Surgery, Huashan Hospital of Fudan University 540166 , Shanghai, China; 4 College of Fundamental Medical Sciences, Guangzhou University of Chinese Medicine, Guangzhou, Guangdong, China; 5 School of Pharmaceutical Sciences, Guangzhou University of Chinese Medicine, Guangzhou, Guangdong, China; Universidad de los Andes, Bogotá, Colombia

**Keywords:** chitinase, synergy, silkworm exuviae, *Beauveria bassiana*, *Bombyx batryticatus*

## Abstract

This study aimed to investigate the biochemical characteristics of a new GH18 chitinase (BbChi3250) derived from *Beauveria bassiana* KW1, which was isolated from *Bombyx batryticatus*. BbChi3250 occurs widely throughout *Beauveria* and *Cordyceps* species and has low sequence identity (≤39.58%) with already-characterized *B. bassiana* chitinases. Biochemical analyses revealed that BbChi3250 is an *endo*-chitinase and shows good activity toward ethylene glycol chitin, with a *k*_cat_/*K*_m_ of 596.00 mL·mg^–1^·s^–1^. Importantly, BbChi3250 demonstrates significant synergy with protease CbPro in hydrolyzing silkworm exuviae, reflected in high degrees of synergy in releasing *N*-acetyl-β-D-glucosamine and tyrosine equivalents. Scanning electron microscopy analysis of enzyme-digested silkworm exuviae confirmed the enzymes' surface ablation activities and mutual synergy. To the best of our knowledge, this is the first report about the synergistic degradation of silkworm exuviae *in vitro* by protease and *B. bassiana*-sourced chitinase, providing new insights into the enzyme’s properties and applications in the degradation of insect cuticles.

**IMPORTANCE:**

*B. bassiana*, an entomopathogenic fungus, is utilized in producing *Bombyx batryticatus*, a traditional Chinese medicine for treating stroke and related symptoms. This is achieved by infecting *Bombyx mori* larvae with *B. bassiana*. Chitin, a key component of the silkworm cuticle, is hydrolyzed by endo-chitinase, a critical virulence factor of *B. bassiana*. Despite the presence of multiple chitinase-encoding genes in *B. bassiana* strains, such as the twenty GH18 genes in ARSEF 2860, only a few have been studied. Further research on these chitinases could elucidate *B. bassiana*’s pathogenic mechanisms and uncover chitinases with new properties.

## INTRODUCTION

The well-known entomopathogenic fungus *Beauveria bassiana* can infect a wide range of insects and has value as a biological control agent in agriculture and forestry. *B. bassiana* is also used to manufacture the economically valuable traditional Chinese medicine *Bombyx batryticatus*, which is used to treat stroke, mouth and eye slanting, facial muscle twitching, or limb numbness in clinical practice (1). This medicine is produced by infecting 4th–5th instar larvae of *Bombyx mori* Linnaeus (commonly known as silkworm) with *B. bassiana*. Infection is typically divided into five stages: contact attachment, germination by repetition, epidermal invasion, colonization growth, and death. Epidermal invasion is a critical step and depends on the hydrolysis and penetration of insect cuticle, which is mainly composed of chitin, proteins, and lipids. In this regard, the pathogenicity of *B. bassiana* is tightly linked to its ability to produce enzymes that degrade insect cuticles (2, 3).

Chitin is a linear polymer consisting of β-(1, 4)-linked *N*-acetyl-β-D-glucosamine (GlcNAc) units and generally makes up 20%–40% of the dry weight of insect cuticles (4). A variety of enzymes, including *endo*-chitinases (EC 3.2.1.14, commonly known as chitinase), *exo*-chitinases (EC 3.2.1.29), chitin deacetylases (EC 3.5.1.41), and lytic polysaccharide monooxygenases (EC 1.14.99.53), are required to completely degrade chitin (5). The most important of these is chitinase, which may randomly cleave β-(1, 4)-glycosidic bonds within the polymer, thereby producing oligomeric and monomeric components. Current categorization using amino acid sequence similarity places chitinases into glycoside hydrolase (GH) families 18, 19, 23, and 48 (6). Most fungal chitinases belong to GH family 18 (GH18), which can be further divided into subfamilies A, B, C, and D. These subfamilies exhibit variations in domain architecture, hydrolytic characteristics, and biological function (7).

Generally, strains of *B. bassiana* harbor multiple chitinases, many of which may have different functions. For example, *B. bassiana* ARSEF 2860 possesses 20 different GH18 chitinase-encoding genes on the genome (8). However, only a few *B. bassiana* chitinases have been studied to date. For example, the Pei group found that Bbchit1 could degrade colloidal chitin (CC) and that overexpression of Bbchit1 in a *B. bassiana* transformant increased fungal virulence toward aphids, resulting in a 34.1% reduction in lethal concentration 50% (LC_50_). Transformant overexpression of chimeric bifunctional protease/chitinase (CDEP1:Bbchit1) led to a 67.4% reduction in LC_50_ compared with wild-type. This decrease was greater than the additive effects of transformant overexpression of CDEP1 (no reduction in LC_50_) and Bbchit1, indicating that protease and chitinase acted synergistically to enhance virulence (9–11). Moreover, the addition of chitinase derived from either *B. bassiana* or *Trichoderma koningiopsis* into *B. bassiana* conidia solution increased its insecticidal efficacy against *Diatraea saccharalis*, which could be attributed to the partial chitinase-induced cuticle degradation in insects following fungal germ tube penetration of the epicuticle (12, 13). Production of *Bombyx batryticatus* requires cuticle penetration by *B. bassiana* conidia; however, no studies have examined silkworm cuticle degradation by *B. bassiana* chitinase or the synergistic degradation that occurs when chitinase and protease act together. In addition to their role as a virulence factor for insect-pathogenic fungi, chitinases with excellent properties have been used in the production of GlcNAc, chitooligosaccharides, and antifungal preparations (5, 14–16). Further study of *B. bassiana* chitinases would thus not only deepen understanding of the mechanism underlying *B. bassiana* pathogenesis but would also contribute to efforts to identify chitinases with new and excellent characteristics.

Recently, a new strain (designated *B. bassiana* KW1) was isolated by our group from *Bombyx batryticatus* and subjected to whole-genome sequencing. In this study, a new putative chitinase gene (*Bbchi3250*), which is highly conserved in various *B. bassiana* strains, was identified on the genome of *B. bassiana* KW1. Then, gene cloning, protein expression, and enzyme characterization were carried out. Importantly, to evaluate the potential application of BbChi3250 for silkworm cuticle degradation and explore its synergistic relationship with protease, BbChi3250 and protease CbPro were used to degrade silkworm exuviae separately or together, and the microstructures of enzyme-digested silkworm exuviae were analyzed using SEM as well. The integration of advanced biochemical characterization with SEM and synergistic degradation assays represents a novel approach to studying chitinase-protease interactions.

## RESULTS AND DISCUSSION

### Gene identification, protein expression, and zymography

**Fig 1:**
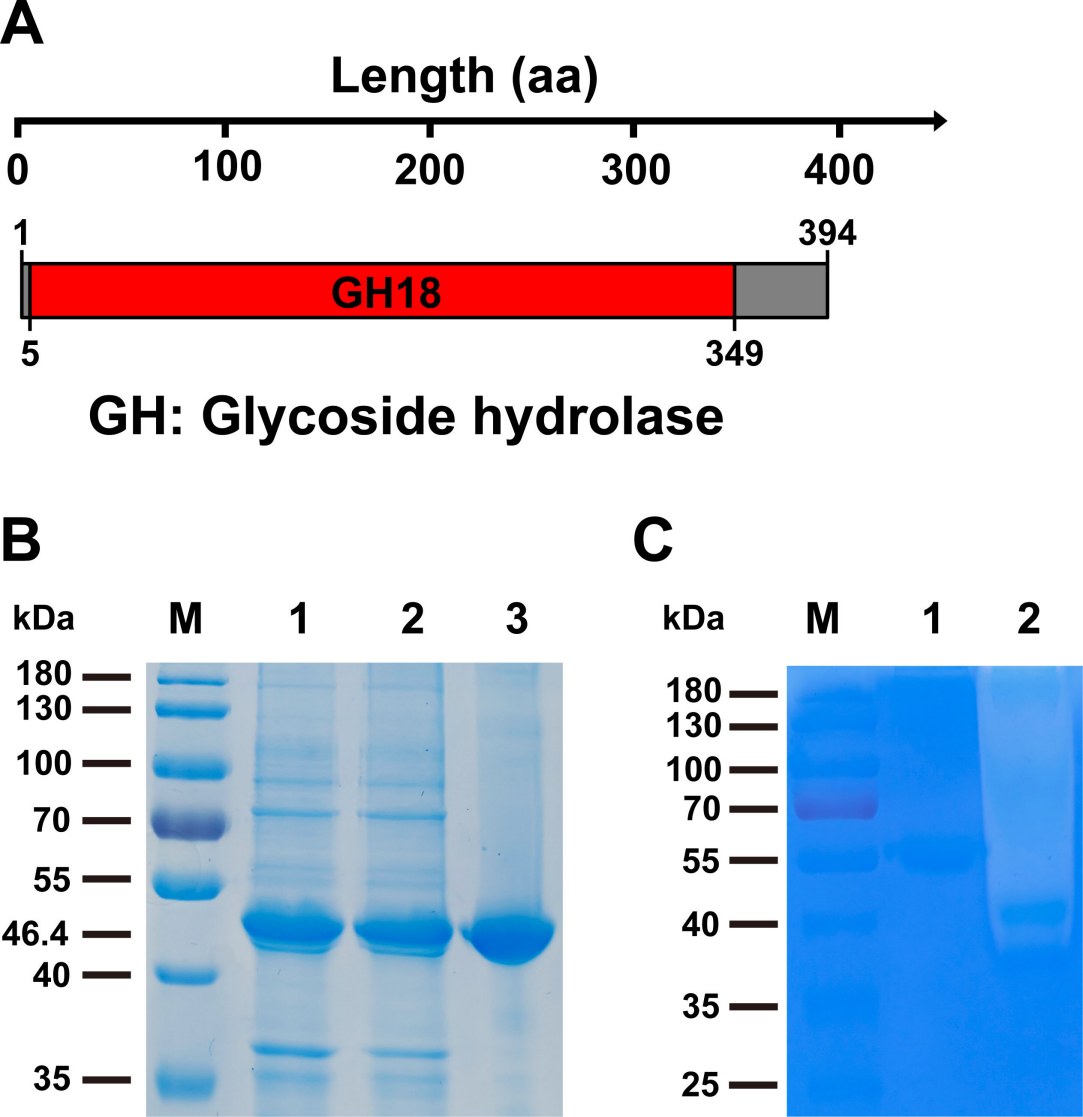
Identification, recombinant expression, and zymography of BbChi3250. (**A**) Domain architecture for BbChi3250. (**B**) SDS-PAGE analysis of BbChi3250. Lane M, protein standards (35‒180 kDa); Lane 1, crude cell lysate of *E. coli*/pET28a-*Bbchi3250* cell cultures; Lane 2, supernatant of the cell lysate of *E. coli*/ pET28a-*Bbchi3250* cell cultures; Lane 3, purified recombinant BbChi3250. (**C**) Ethylene glycol chitin**-**zymography qualitative analysis of BbChi3250. Lane M, protein standards (35‒180 kDa); Lane 1, BSA; Lane 2, BbChi3250.

Genome annotation of *B. bassiana* KW1 revealed the existence of a new putative chitinase gene (*Bbchi3250*), consisting of 1,318 bp and containing two introns. The deduced coding sequence for BbChi3250 (referring to the mature protein) is 1,185 bp, encoding a polypeptide with 394 amino acid residues. A CD search revealed that BbChi3250 possessed a single GH18 catalytic domain (residues 5‒349) (Fig. 1A).

A BLASTp search of GenBank indicated that BbChi3250 showed high sequence identities (>90%) with uncharacterized putative chitinases across various *Beauveria* species (*B. bassiana* D1-5, enzyme accession number: KGQ03302, 100%; *B. bassiana* ARSEF 2860, AIT18880, 99.71%; *B. bassiana* ERL836, KAF1734293, 99.24%; *B. bassiana* JEF 350, KAH8709201, 99.21%; *B. bassiana* JEF-007, PMB72293, 98.98%; *B. asiatica*, KAK8148978, 95.1%; *B. brongniartii*, OAA50774, 94.81%; and *B. bassiana* ARSEF 2860, XP_008602626, 90.61%) and *Cordyceps* species (*C. farinose*, AIU45788, 100%; *C. javanica*, TQV92938, 90.86%; *C. fumosorosea* ARSEF 2679, XP_018702257, 90.86%; *C. cicadae*, QDJ94326, 90.61%; *C. fumosorosea*, ACP40175, 90.61%). Additionally, orthologous proteins of BbChi3250 have been identified in a recently reported *B. bassiana* strain (17), and the number of these orthologs would increase as the number of sequenced genomes grows. In comparison with characterized chitinases, BbChi3250 exhibited maximum similarity with rMvEchi (GenBank accession number: QEP29042, 71.57% identity) from *Myrothecium verrucaria* (18), followed by AnChiB (RDH18539, 59.60%) from *Aspergillus niger* (19), VlCHI (AGU42402, 51.67%) from *Verticillium lecanii* (20), Chit42 (AAB34355, 50.40%) from *Trichoderma harzianum* (14), Bbchit1 (AAN41259, 39.58%) from *B. bassiana* Bb0062 (10), and RmchiA (7FBT_A, 25.90%) from *Rhizomucor miehei* (Table 1) (21). These findings reveal that BbChi3250 is a new putative chitinase that is highly conserved in *Beauveria* and *Cordyceps* species, indicating that this enzyme may play an essential role in these strains, and its enzymatic properties are worthy of further study.

**TABLE 1:** Comparison of BbChi3250 with its homologous GH18 chitinases^*e*^

Enzyme^*a*^, source	Length (aa)^*b*^, identity^*c*^	pH & temp optima	Specific activity (U·mg^−1^)	Km (mg·mL^−1^)	Kcat (s^−1^)	Kcat/Km (mL·mg^−1^·s^−1^)	Reference
BbChi3250, *Beauveria bassiana* KW1	394, –^*f*^	pH 5.0, 30–35°C	8.10 ± 0.04 (EGC) 4.43 ± 0.01 (CC) 0.90 ± 0.04 (acid-treated silkworm exuviae) 0.20 ± 0.06 (powdered chitin) 0.02 ± 0.02 (powdered silkworm exuviae)	3.18 ± 0.21(CC) ± 0.00(EGC)	6.53 ± 0.13(CC) 5.96 ± 0.12(EGC)	2.05 ± 0.14 (CC) 596.00 ± 12.00 (EGC)	This study
rMvEchi, *Myrothecium verrucaria*	394, 71.57%	pH 7.0, 30°C	1.12 ± 0.10 (acid swollen chitin) 0.35 ± 0.02 (EGC) 0.90 ± 0.04 (acid swollen chitosan)	2.05 × 10^−3^	266.54 ± 3.71	129.83 × 10^3^ (4-methylumbelliferyl-*N,N',N''*-triacetyl-beta-chitotrioside)	(18)
AnChiB, *Aspergillus niger*	446, 59.60%	pH 6.0, 40°C	28.08 ± 0.62 (EGC) 9.62 ± 0.20 (α-chitin) 14.41 ± 0.29 (β-chitin) 6.41 ± 0.58 (mycelial waste)	1.24	2.07	1.66 (EGC)	(19)
VlCHI, *Verticillium lecanii*	423, 51.67%	pH 4.6, 40°C	29.26 (CC)	NA^*d*^	NA^*d*^	NA^*d*^	(20)
Chit42, *Trichoderma harzianum*	423, 50.40%	pH 6.0, 35°C	5.20 (CC)	1.70 ± 0.10	5.00 ± 0.10	2.94 ± 0.18	(14)
Bbchit1, *Beauveria bassiana* Bb0062	348, 39.58%	pH 5.0–6.0, 55°C	4.00 (CC)	NA^*d*^	NA^*d*^	NA^*d*^	(10)
RmChiA, *Rhizomucor miehei*	378, 25.90%	pH 5.0, 50°C	0.47 ± 0.02 (CC) 0.68 ± 0.03 (N-chitooligosaccharides) 0.64 ± 0.01 (EGC)	3.73 ± 0.09	0.53 ± 0.02	0.14 ± 0.01 (CC)	(21)

^*a*^ Enzyme accession numbers for the enzymes are as follows: BbChi3250, XCA85047; rMvEChi, QEP29042; AnChiB, RDH18539; VlCHI ,AGU42402; Chit42, AAB34355; Bbchit1, AAN41259. The accession number for RmChiA is not available.

^*b*^ Mature protein length.

^*c*^ Sequence identities between BbChi3250 and the other proteins were calculated using the CLUSTAL W server (https://www.genome.jp/tools-bin/clustalw).

^*d*^ NA, the information was not available.

^*e*^ Among the data related to specific activities and kinetic parameters, some publications provided average values only.

^*f*^ –, no data.

The SDS-PAGE analyses revealed that the cell lysate supernatant and the crude cell lysate of *Escherichia coli*/pET28a-*Bbchi3250* was nearly identical, and the purified protein’s band size conformed to the predicted molecular mass of recombinant BbChi3250 (46.4 kDa), indicating that recombinant BbChi3250 was expressed in a soluble form and purified to homogeneity (Fig. 1B). Zymography is an intuitive approach for visualizing hydrolytic activity on the basis of substrate degradation (16). As shown in Fig. 1C , a lytic zone developed by purified Bb3250 was visualized in natural light, whereas nothing was detected around the BSA band, suggesting that BbChi3250 exhibited chitinase activity. Activity assay showed that the cell lysate supernatant had a specific activity of 1.98 U·mg^–1^ toward CC, whereas purified BbChi3250 had a higher specific activity of 4.43 U·mg^–1^, representing a 2.24-fold purification (Table 2). It follows from the above that the heterologous expression of fungal enzyme genes in *E. coli* is a practical approach to obtain a soluble enzyme with appropriate function.

**TABLE 2:** Purification stages of BbChi3250 from *E. coli/*pET28a-*Bbchi3250*

Step of purification	Full of protein (mg)^*a*^^,*b*^	Specific activity (U·mg^–1^)^*a*^^,*c*^	Purification factor (*n*-fold)
Supernatant of cell lysate (250 mL)	55.89 ± 4.37	1.98 ± 0.01	1
Purification by TALON metal affinity resins	1.78 ± 0.10	4.43 ± 0.01	2.24

^*a*^ Assays were replicated three times. Data are presented as mean ± standard deviation.

^*b*^ Protein concentration of BbChi3250 was evaluated using BCA protein assay kits (Tiangen, Beijing, China).

^*c*^ BbChi3250 units are defined as the quantity of chitinase liberating 1 µmol of GlcNAc equivalents from colloidal chitin per min at pH 5.0 and 30°C.

**Fig 2:**
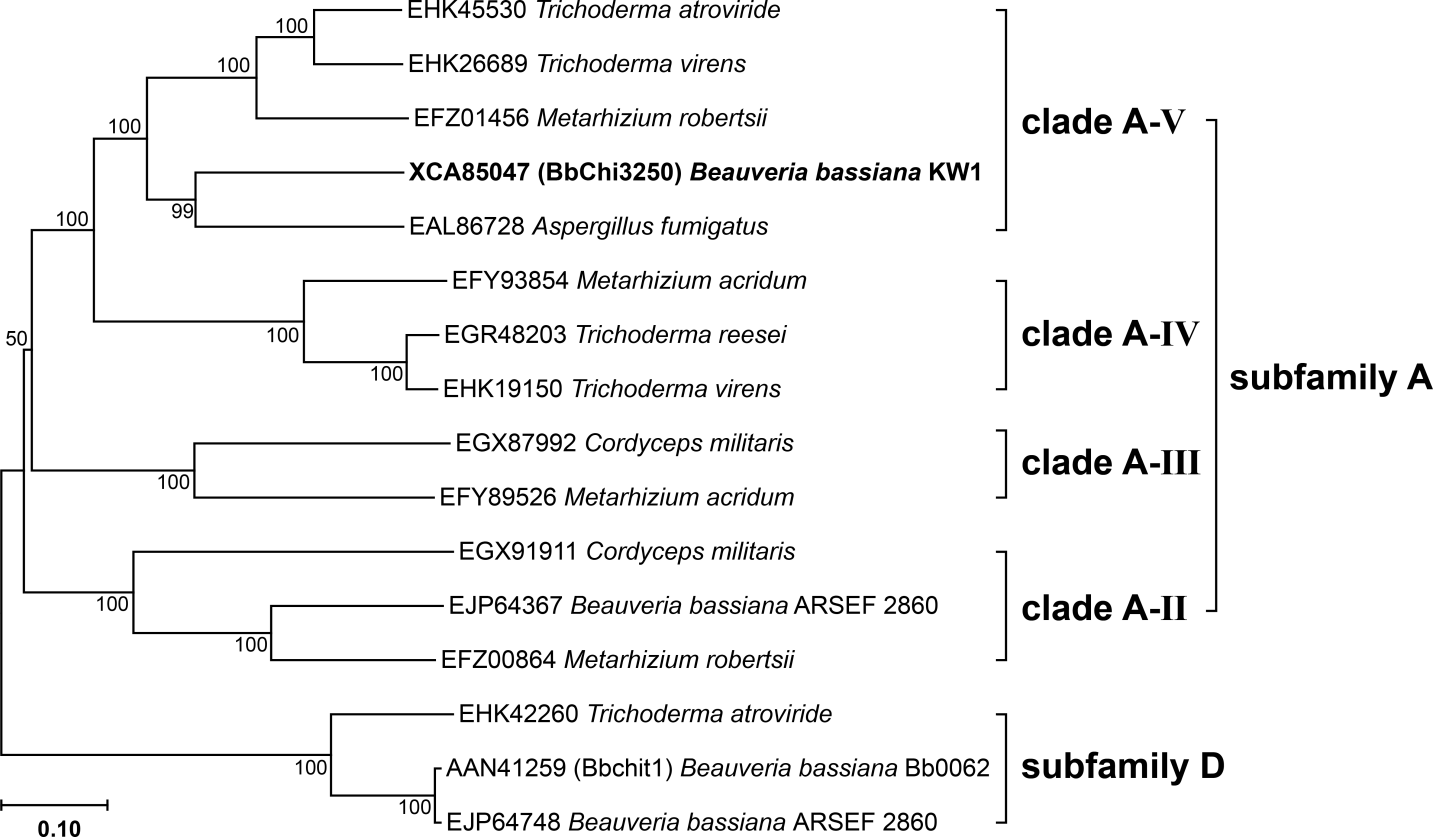
Phylogenetic analysis of BbChi3250 and closely related GH18 fungal chitinases. Except for BbChi3250, all amino acid sequences were obtained from GenBank. The neighbor-joining (1,000 bootstraps) phylogenetic tree was constructed using MEGA 11.0 software following ClustalW alignment. The scale bar at the bottom represents 1,000 amino acid substitutions, and the value near each branch indicates the reliability percentage in the bootstrap test of that branch.

**Fig 3:**
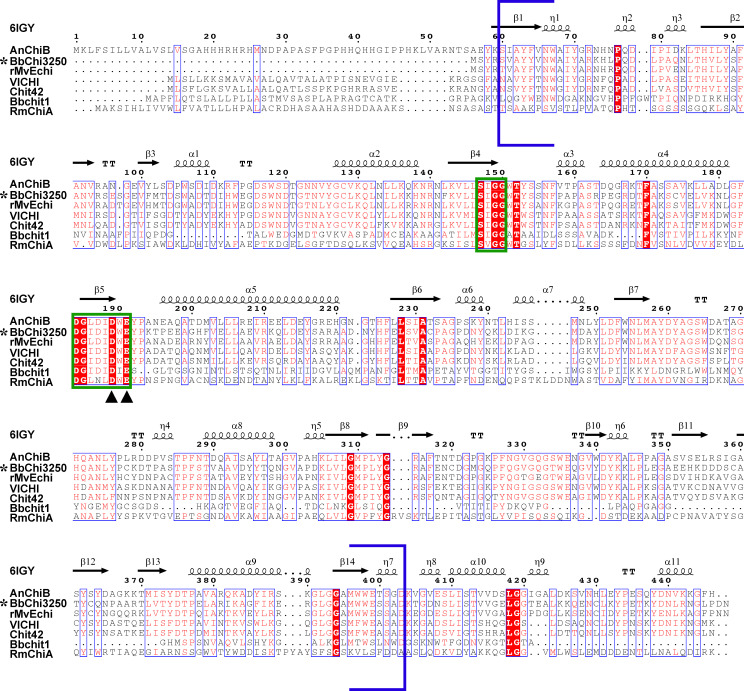
Multiple sequence alignment of BbChi3250 and closely characterized homologs. All protein sequences were retrieved from GenBank or Protein Data Bank. The proteins (accession numbers) and organisms are: BbChi3250 (XCA85047), *B. bassiana* KW1; AnChiB (RDH18539), *Aspergillus niger* (19); rMvEChi (QEP29042), *Myrothecium verrucaria* (18); VlCHI (AGU42402), *Verticillium lecanii* (20); Chit42 (AAB34355), *Trichoderma harzianum* (14); Bbchit1 (AAN41259), *B. bassiana* Bb0062 (10); RmChiA (7FBT), *Rhizomucor miehei* (21). The crystal structure of AnChiB (6IGY) was used as a structure template. α-Helices, β-strands, and strict β-turns were rendered as squiggles, arrows, and TT. Conserved residues are indicated by red background shading. Sequences enclosed in blue brackets are GH18 domains. The conserved motifs SXGG and DXXDXDXE are indicated by the green box, with crucial active site residues indicated by black triangles.

Under current chitinase phylogenies, chitinases in subfamilies B and C generally contain a carbohydrate-binding module (CBM) in addition to a catalytic domain, whereas GH18 chitinases with a single catalytic domain have been identified in subfamilies A and D. Furthermore, subfamily A can be further divided into A-II, A-III, A-IV, and A-V clades based on the presence or absence of signal peptides and differences in conserved motifs (22). Because BbChi3250 does not possess a CBM, it cannot be assigned to subfamilies B and C. To estimate the evolutionary relatedness of BbChi3250 and its homolog Bbchit1 to other chitinases, many fungal chitinases from subfamilies A and D were used to construct a phylogenetic tree, in which BbChi3250 was assigned into clade A-V of subfamily A, whereas Bbchit1 was classified into subfamily D (Fig. 2). Sequence analysis revealed that BbChi3250 contained highly conserved SXGG (SIGG) and DXXDXDXE (DGLDIDWE) motifs, which are associated with substrate-binding and catalysis, respectively (Fig. 3). Moreover, the third Asp (D) from the left and the Glu (E) in the DXXDXDXE motif have been identified as residues crucial for chitinase activity (7).

### Effect of pH, temperature, and substrate on Bb3250 performance

**Fig 4:**
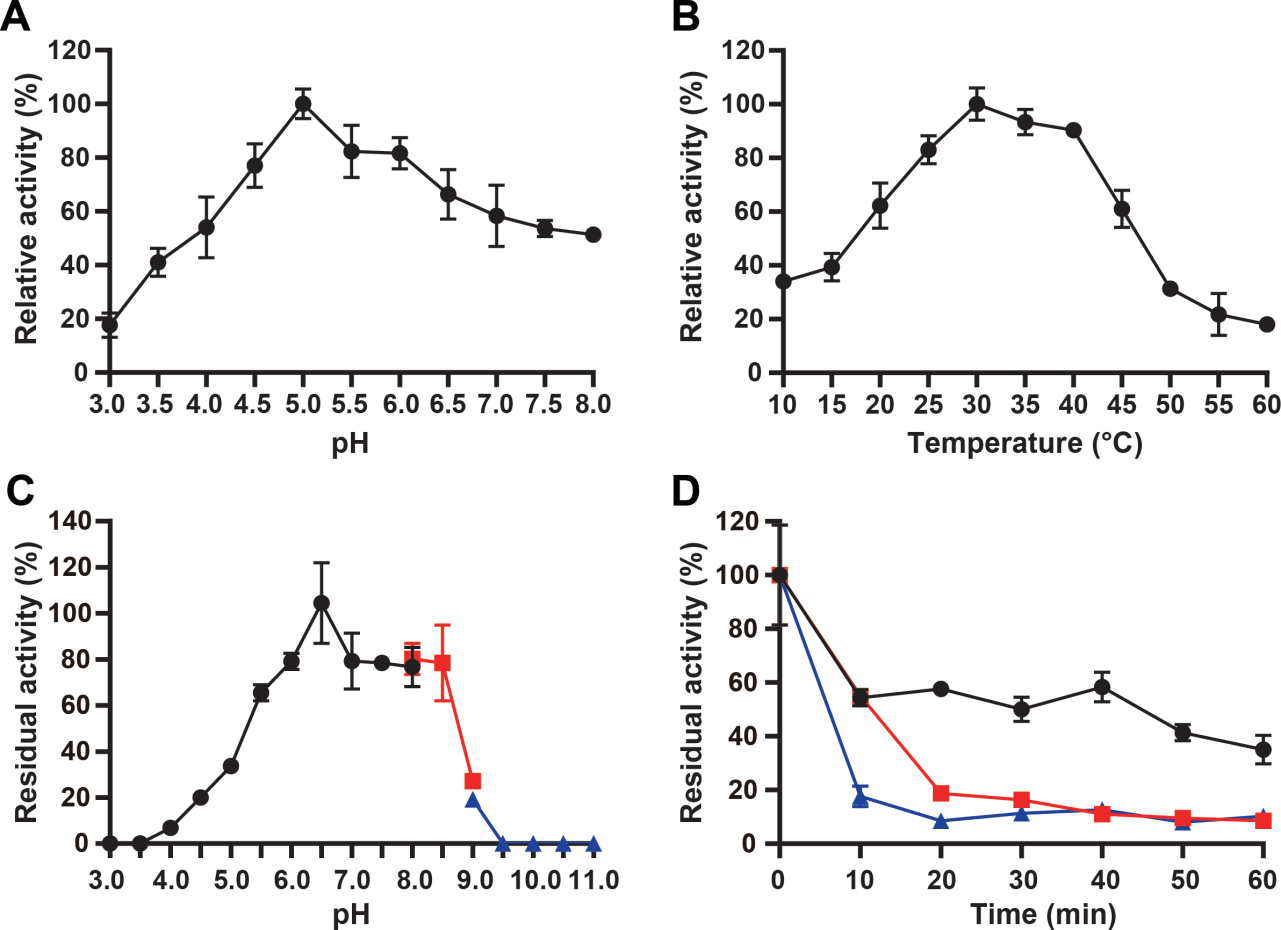
Influence of pH and temperature on BbChi3250. (**A**) pH profiles of BbChi3250. (**B**) Temperature profiles of BbChi3250. (**C**) pH stability analysis of BbChi3250. (**D**) Thermostability analysis of BbChi3250. Error bars in panels A, B, C, and D indicate the standard deviations of triplicate reactions. Symbols for pH stability in panel C: black circles, 100 mM Na_2_HPO_4_–citric acid buffer (pH 3.0-8.0); red squares, 100 mM Tris-HCl buffer (pH 8.0-9.0); blue triangles, 100 mM glycine-NaOH buffer (pH 9.0-11.0). Symbols for thermostability in panel D: black circles, 30°C; red squares, 35°C; blue triangles, 40°C.

The optimal reaction pH for recombinant BbChi3250 was determined to be pH 5.0, with over 60% of the highest activity retained between 4.5 and 6.5, indicating the enzyme functions well under weakly acidic conditions (Fig. 4A). Optimal temperature determination revealed that enzyme activity was highest at 30°C–35°C, with more than 80% of the enzyme’s highest activity measured at temperatures between 25°C and 40°C (Fig. 4B). The pH stability profile indicated that recombinant BbChi3250 maintained over 75% relative activity at pH 6.0–8.5 (Fig. 4C). Subsequent thermal stability analysis showed that recombinant BbChi3250 retained approximately 40% of its initial activity following incubation at 30°C for 50 min and retained less than 20% of its original activity after incubating for 20 min at 35°C and 10 min at 40°C, respectively. (Fig. 4D). These findings indicate that BbChi3250 is a thermolabile acidic enzyme with an optimal temperature consistent with its host strain *B. bassiana* KW1 (optimal growth temperature: 25‒30°C. Data for all available homologs indicate that these chitinases are acidic, with optimal temperatures ranging from 30 °C to 40 °C for chitinases from *M. verrucaria*, *T. harzianum*, *A. niger*, and *V. lecanii*, identical or similar to the optimum temperature for BbChi3250. However, RmchiA from *R. miehei* was most active at 50°C, and Bbchit1 from *B. bassiana* Bb0062 had the highest optimal temperature (55°C) (Table 1). Although the enzymes listed above are acidic and mesophilic or cold-active, chitinases with varying pH and temperature optima have been identified in GH18, including alkaline and thermophilic chitinases. For example, the marine metagenome-derived chitinase Chi304 displayed maximum activity at pH 9.0 and 85°C (23).

**Fig 5:**
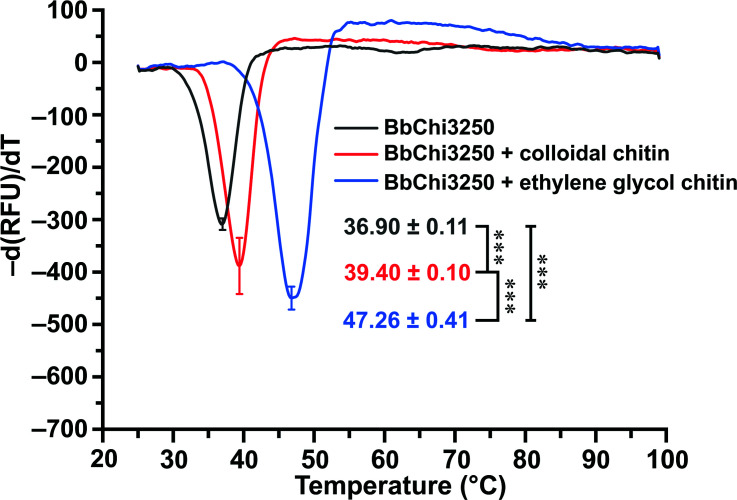
Protein thermal shift assay of BbChi3250 in the presence or absence of substrate (CC or EGC). Each assay was repeated three times, and *T*_m_ values are presented as mean ± standard deviation; *** very significant difference (*P* < 0.001).

The *T*_m_ for BbChi3250 was determined to be 36.90°C, which may explain its thermolability when pretreated at 35°C or 40°C. However, the addition of CC increased *T*_m_ to 39.40°C, whereas the supplementation of EGC substantially increased *T*_m_ to 47.26°C (Fig. 5). The corresponding δ*T*_m_ values were +2.5°C and +10.36°C, suggesting that BbChi3250 may have a higher binding affinity for EGC.

### Analyses of substrate specificity, hydrolysates, kinetic parameters, and three-dimensional structure models

To determine substrate specificity for BbChi3250, a series of polysaccharides was tested. As shown in Table 1, the specific activity toward EGC was highest (8.10 U·mg^–1^), followed in the descending order by CC (4.43 U·mg^–1^), acid-treated silkworm exuviae (0.90 U·mg^–1^), powdered chitin (0.20 U·mg^–1^), and powdered silkworm exuviae (0.02 U·mg^–1^). Remarkably, the specific activity of BbChi3250 toward EGC was higher than that toward CC, similar to Sschi28 from *Streptomyces sampsonii*, whose specific activity toward EGC was nearly 22-fold higher (222.3 U·mg^–1^) than toward CC (20.1 U·mg^–1^) (24). However, many chitinases prefer CC (25–28). For example, PsChi82 from *Paenibacillus shirakamiensis* exhibits the highest activity toward CC (12.0 U·mg^–1^), but its specific activity toward EGC is much lower (3.0 U·mg^–1^) (25).

Moreover, BbChi3250 demonstrated hydrolytic activity against recalcitrant substrates chitin and silkworm exuviae, indicating that the enzyme may facilitate insect infection by *B. bassiana* KW1. Our group has shown that *B. bassiana* KW1 is highly virulent to *Bombyx mori* Linnaeus, with a fatality rate of 98% when inoculating the 5th instar larvae with conidia at a concentration of 5 × 10^6^. We infer that BbChi3250 may represent part of its virulence factor, which would await further confirmation. To the best of our knowledge, this is the first time that silkworm exuviae have been used as a substrate to characterize chitinase. *B. bassiana* was first isolated by Agostino Bassi in 1834 and identified as the cause of the devastating muscardine disease of silkworms (8), whereas silkworm cadavers are regarded as a valuable traditional Chinese medicine*, Bombyx batryticatus*. *B. bassiana* species promotes the infection of silkworms or other insects by producing a chitinase-containing enzyme complex to degrade the silkworm epidermis. Therefore, the characterization of *B. bassiana*-sourced chitinase using silkworm exuviae as a substrate is novel, but it is basically consistent with the actual process of *Bombyx batryticatus* production and biological pest control.

As presented in Table 1, the *K*_m_, *k*_cat_, and *k*_cat_/*K*_m_ for BbChi3250 on CC were 3.18 mg·mL^–1^, 6.53 s^–1^, and 2.05 mL·mg^–1^·s^–1^, respectively; the *K*_m_, *k*_cat_, and *k*_cat_/*K*_m_ for BbChi3250 on EGC were 0.01 mg·mL^–1^, 5.96 s^–1^, and 596.00 mL·mg^–1^·s^–1^, respectively. This indicates that BbChi3250 has a much higher affinity for EGC than CC, which well explains the above differences in δ*T*_m_. Some of the homologs of BbChi3250 have been kinetically analyzed; the substrates used include 4-methylumbelliferyl-*N,N',N''*-triacetyl-beta-chitotrioside, CC, and EGC. However, the reaction conditions for measuring kinetic parameters are not exactly the same. When comparing the catalytic efficiency of different enzymes, it is necessary to refer to their *k*_cat_/*K*_m_ values for the same substrate and the reaction conditions. For example, the *k*_cat_/*K*_m_ of AnChiB toward EGC was determined to be 1.66 mL·mg^–1^·s^–1^, using 0.5 µM enzyme under its optimal pH and temperature (pH 6.0, 40°C). In comparison, BbChi3250 showed a *k*_cat_/*K*_m_ of 596.00 mL·mg^–1^·s^–1^ for EGC under its optimal reaction pH and temperature (pH 5.0, 30°C), indicating that BbChi3250 exhibits much higher catalytic efficiency at a lower reaction temperature. Since chitin is the second most abundant natural biopolymer after cellulose and is found in a wide range of organisms, including insects, plants, bacteria, fungi, and mammals. However, the water insolubility and crystalline tertiary structure of chitin make it difficult to access and degrade by chitinase (4). To increase the solubility and substrate surface, chitin can be processed with hydrochloric acid into CC or ethylene glycol into EGC. Therefore, compared with powdered chitin, chitinase generally exhibits higher activity toward EGC and CC, as observed from BbChi3250. In addition, the specific activity of BbChi3250 toward EGC was higher than that toward CC, which may be due to the higher solubility and substrate accessible surface area of EGC. Chitinases with good activity have the potential to convert chitin substrates into highly profitable derivatives such as chitosan and chitooligosaccharides (5, 23). The high activity of BbChi3250 toward EGC makes it an interesting enzyme for the biotransformation of EGC into high-value derivatives, thus demonstrating promising application prospects.

**Fig 6:**
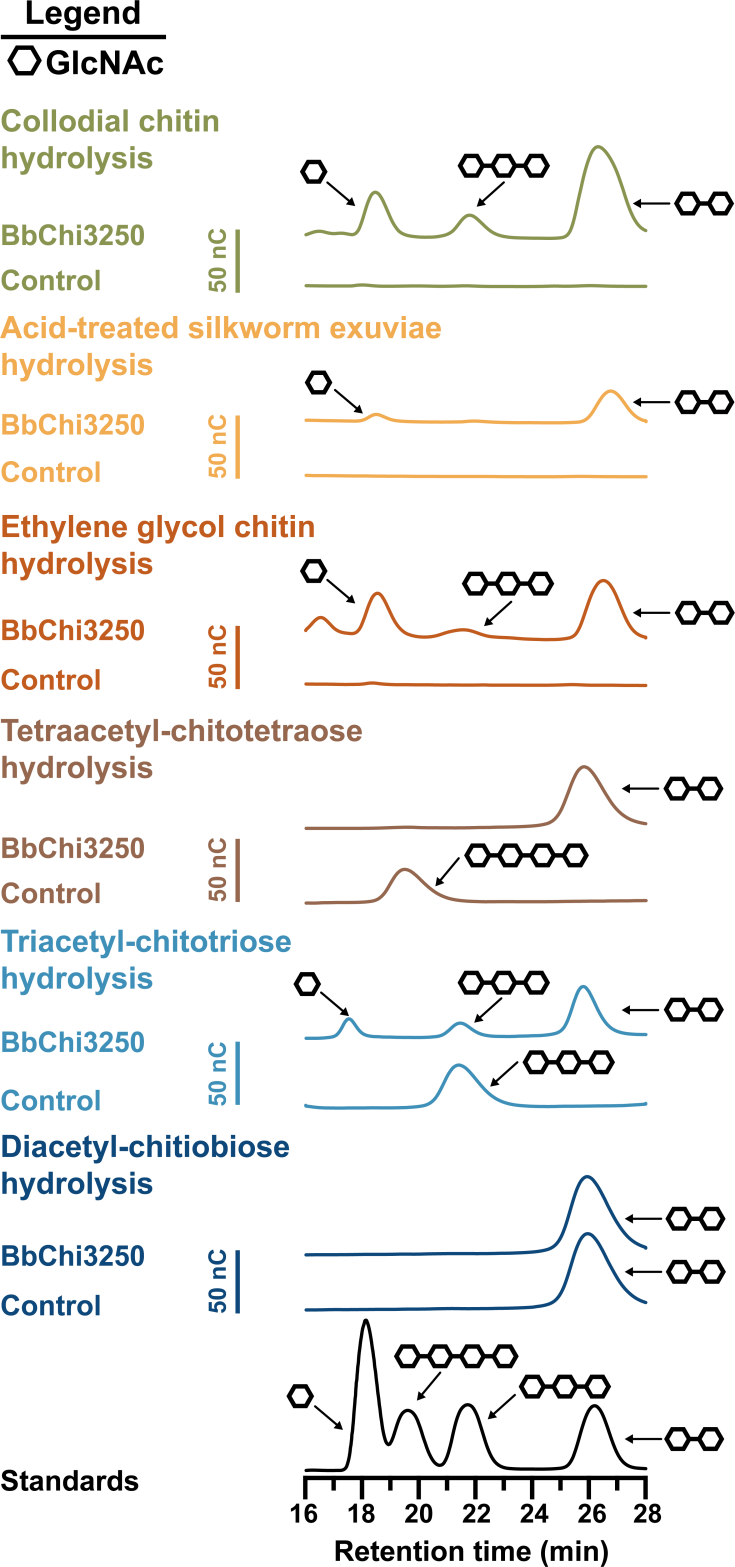
HPAEC-PAD analyses of the hydrolysates of colloidal chitin, acid-treated silkworm exuviae, ethylene glycol chitin, and chitooligosaccharides [diacetyl-chitobiose (GlcNAc)_2_, triacetyl-chitotriose (GlcNAc)_3_, and tetraacetyl-chitotetraose (GlcNAc)_4_] by BbChi3250. The pulsed amperometric detection (PAD) response was measured in nanocoulombs (nC).

To investigate the mode of hydrolytic action, we analyzed the hydrolysates of CC, acid-treated silkworm exuviae, EGC, and chitooligosaccharides (from 2 to 4 GlcNAc units) using HPAEC-PAD. As shown in Fig. 6, GlcNAc, (GlcNAc)_2_, and a small amount of (GlcNAc)_3_ were released from CC and EGC by BbChi3250, whereas only GlcNAc and (GlcNAc)_2_ were produced from acid-treated silkworm exuviae. Additionally, BbChi3250 hydrolyzed (GlcNAc)_4_ into (GlcNAc)_2_ and released (GlcNAc)_2_ and GlcNAc from (GlcNAc)_3_. However, it did not hydrolyze (GlcNAc)_2_. These findings imply an endo-type cleavage pattern for BbChi3250. In addition to *endo*-chitinase, some members in GH18 have been identified as *exo*-chitinase (29), bifunctional *endo*-chitinase/*exo*-chitinase (13, 23), bifunctional chitobiosidase/*N*-acetyl-D-glucosaminidase (15), and bifunctional *endo*-chitinase/chitosanase (25). Surprisingly, the elution order of saccharides did not correspond to the degree of polymerization, a phenomenon that has been previously reported. This could be because the acidic hydroxyl groups (the 2-OH of glucose moieties) in chitooligosaccharides or chitin were not 100% acetylated, but hydrolysates were eluted with alkaline solution (20 mM NaOH), resulting in varying degrees of substitution and acid-base reaction (14, 30). Additionally, differences in the interaction mode between the oligosaccharides and the column matrix may also serve as a potential factor influencing the elution order.

**Fig 7:**
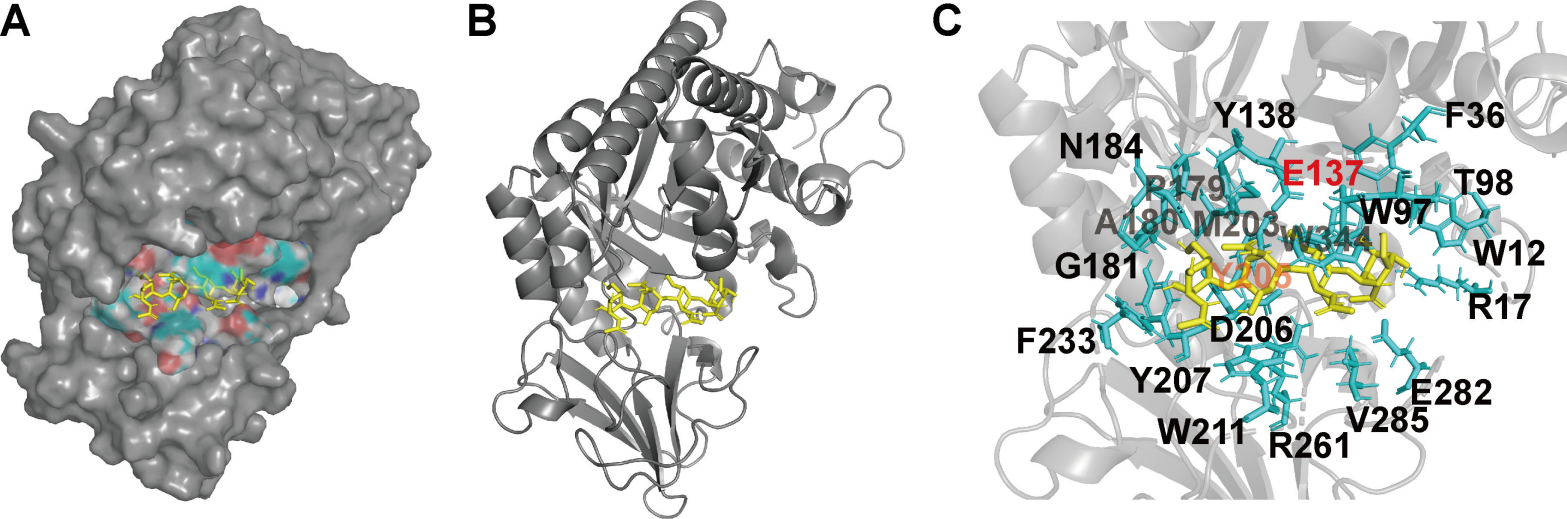
The modeled molecular surface (**A**), ribbon diagram (**B**), and active site three-dimensional representation (**C**) of the BbChi3250-(GlcNAc)_4_ complex. (GlcNAc)_4_ is presented in yellow.

Prediction of the tertiary structure of BbChi3250 reveals the existence of a typical TIM barrel (β/α)_8_, a characteristic feature that occurs widely in GH18 chitinases (Fig. 7B) (7). The predicted BbChi3250-(GlcNAc)_4_ complex structure indicates that the substrate is embedded within a deep, semi-closed tunnel (Fig. 7A and B), and enzyme-substrate interaction analysis revealed that 21 residues (N184, Y138, F36, P179, E137, T98, A180, M203, W344, W97, W12, G181, Y205, R17, F233, D206, Y207, W211, R261, V285, and E282) were within ≤4.0 Å of (GlcNAc)_4_ and potentially interacted with substrate via hydrogen bonding (Fig. 7C). Additionally, residues E315 and Y390 of ChiA from *Serratia marcescens* have been shown to play a central role in the hydrolysis of (GlcNAc)_4_ (PDB code: 1K9T) (31). Structural superimposition revealed that residues E137 and Y205 of the BbChi3250-(GlcNAc)_4_ complex correspond to E315 and Y390 of ChiA, respectively, suggesting that residues E137 and Y205 were involved in catalysis. However, the speculation awaits confirmation by future mutational research. Given the differences in amino acid sequence and enzymatic properties between BbChi3250 and other characterized enzymes, as well as the presence of the GH18 catalytic domain, BbChi3250 is identified as a new GH18 chitinase.

### Synergistic hydrolysis of silkworm exuviae

**Fig 8:**
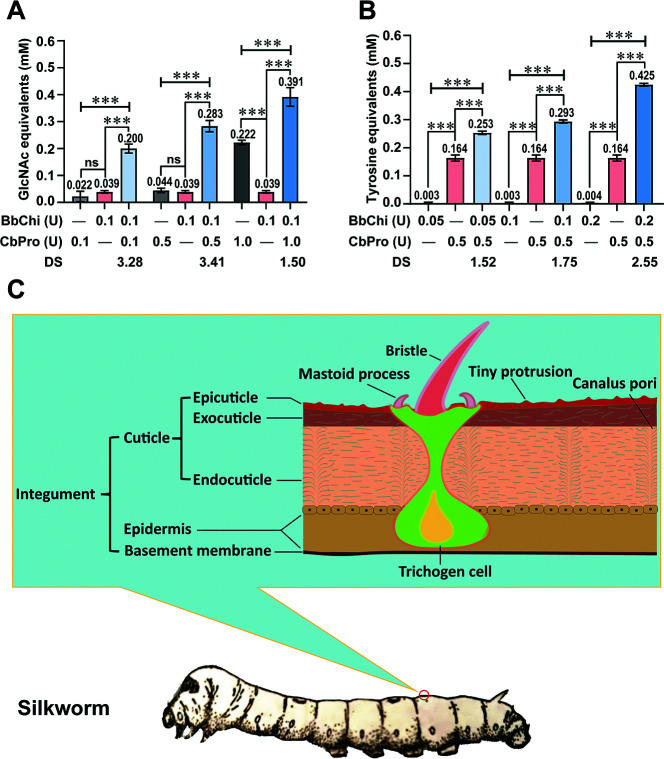
Synergistic hydrolysis of silkworm exuviae by BbChi3250 and CbPro. (**A**) Synergistic effect of CbPro on the degradation of powdered silkworm exuviae by BbChi3250. (**B**) Synergistic effect of BbChi3250 on the degradation of powdered silkworm exuviae by CbPro. (**C**) Schematic diagram of the silkworm body wall. The digit upon each bar is the mean value of triplicate reactions, with error bars representing standard deviation. BbChi refers to Bbchi3250, *** very significant difference (*P* < 0.001), ** significant difference (0.001 < *P* < .01), and * statistical difference (0.01 < *P* < .05).

Silkworms are larvae of *Bombyx mori* and molt four times during the larval stage, which is thus divided into five instar phases. During each molting process, a new cuticle is generated and the old cuticle is shed, producing silkworm exuviae (32). *Bombyx batryticatus* is the dried dead silkworm prepared by infecting the 4th–5th instar larvae using a lethal *B. bassiana* strain, against which the cuticle is the first barrier to infection. As illustrated in Fig. 8C, the silkworm cuticle is comprised of thin epicuticle, exocuticle, and endocuticle layers from the outermost to the innermost, where exocuticle and endocuticle are chief constituents and formed of a chitin-protein complex (33). Additionally, the protein, chitin, and fat proportions in tasar silkworm exuviae were approximately 62%, 11.52%, and 3.9%, respectively (34). In this regard, we speculate that chitinase and protease may synergistically promote cuticle degradation.

In the hydrolysis of powdered silkworm exuviae, the GlcNAc equivalents produced by 0.1 U BbChi3250, 0.1 U CbPro, 0.5 U CbPro, and 1.0 U CbPro were 0.039, 0.022, 0.044, and 0.222 mM, respectively. Surprisingly, although protease cannot degrade chitin, the amount of GlcNAc equivalents produced by 1.0 U CbPro was significant, whereas 0.1 U CbPro or 0.5 U CbPro produced only negligible amounts of GlcNAc equivalents. More importantly, the addition of 0.1, 0.5, or 1.0 units of CbPro to the 0.1 U BbChi3250 treatment increased the hydrolysis of powdered silkworm exuviae, increasing concentrations of GlcNAc equivalents to 0.2, 0.283, and 0.391 mM, respectively. Resulting DS values were 3.28, 3.41, and 1.50, respectively (Fig. 8A, the detailed data were presented in Table S1 of the Supporting Information). On the other hand, 0.164 mM tyrosine equivalents were released by 0.5 U CbPro alone, whereas very few tyrosine equivalents were produced by BbChi3250. However, adding 0.05, 0.1, or 0.2 units of BbChi3250 increased tyrosine equivalent concentrations to 0.253, 0.293, and 0.425 mM, respectively. The resultant DS values were 1.52, 1.75, and 2.55, respectively (Fig. 8B; Table S2). These results indicated that BbChi3250 and CbPro have significant synergistic interactions with each other in the degradation of silkworm exuviae, but that optimal dosage ratios of BbChi3250 and CbPro are different for cuticular chitin (1:5) and protein (2:5) degradation. The DS values, together with enzymatic parameters, quantitatively and clearly reflect the enzyme characteristics of BbChi3250, making it easy for researchers to accurately understand and compare.

Previously, *B. bassiana*-sourced chitinase and protease have been shown to enhance the infection efficacy of *B. bassiana* toward green peach aphid *Myzus persicae*, both individually and cooperatively (9, 11, 35). Previous work has used i*n vitro* studies to show that the proteases and chitinases produced by *Conidiobolus coronatus* and *Metarhizium anisopliae* act synergistically to hydrolyze cockroaches and locust cuticles, respectively (36, 37). The fact that infection by *B. bassiana* is lethal to silkworms suggests that the infection process involves cuticle-degrading enzymes. As such, we speculate that BbChi3250, with its wide distribution across *B. bassiana* species, can degrade silkworm exuviae by itself or synergistically with other enzymes. However, to the best of our knowledge, this is the first report of an investigation of the synergistic degradation of silkworm exuviae *in vitro* by a *B. bassiana*-sourced chitinase and another cuticle-degrading protease. Structurally, chitin filaments contribute to the rigidity of silkworm exuviae and serve as scaffolding for cuticular protein attachment, thus forming chitin-protein complexes. It is apparent that most of the chitin filaments in silkworm exuviae are accessible only after the protein matrix is deconstructed, which may explain why only a small amount of GlcNAc equivalents was released from silkworm exuviae when treated with BbChi3250 alone. The probable reason for the considerable amount of GlcNAc equivalents produced by 1.0 unit of CbPro is that high doses of protease degraded a sufficient proportion of the cuticular proteins surrounding chitin filaments to expose the reducing ends of chitin nanofibers. It is then easy to understand the synergy between protease and chitinase. After CbPro hydrolyzed cuticular proteins attached to chitin filaments, more chitin nanofibers were exposed and then degraded by BbChi3250; in return, BbChi3250 may deconstruct the chitin-protein complexes to some extent via the hydrolysis of chitin skeletons, thereby promoting protein degradation by increasing protein accessibility by CbPro, and the cycle repeats. However, this hypothesis awaits experimental verification in the future. It is worth mentioning that *in vitro* experimental conditions may not fully replicate the natural infection process of *B. bassiana* on silkworms, which may affect the synergistic effect of enzymes. Comparing the insecticidal efficacy of *B. bassiana* conidia solution against silkworm larvae in the absence or presence of BbChi3250 and CbPro would further validate the synergistic effect of enzymes. During the hydrolysis process of multicomponent biopolymers, the structure and composition of the biopolymer determine the enzyme complex formula, including enzyme types and dosages in the enzyme complex. The cooperative effect of two or more enzymes is always affected by many factors, including substrate loading, enzyme loading, the dosage ratio of the enzyme members, and reaction time. Enzyme overdosage may even cause these mutualistic effects to shift from synergy to anti-synergy (38–40). It should be noted that although only silkworm exuviae and a few chitin substrates were used to characterize enzyme activity, testing BbChi3250 and CbPro on a broader range of natural substrates would enhance the generalizability of this study, as chitin and protein are the main components of many insect cuticles.

### Time course analysis of the effects of BbChi3250 combined with a commercial protease on the hydrolysis of silkworm exuviae

**Fig 9:**
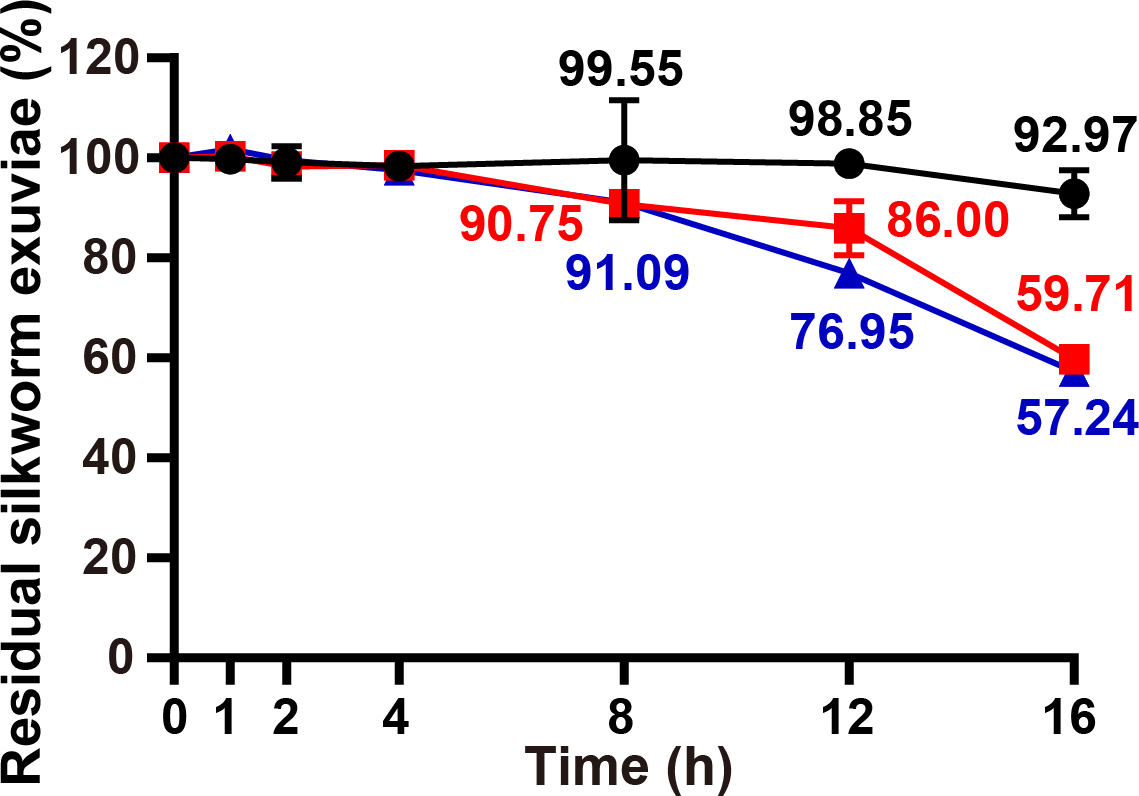
Time course analysis of the effects of BbChi3250 combined with a commercial protease on the hydrolysis of silkworm exuviae. Black circles, silkworm exuviae treated by 0.1 U BbChi3250; red squares, silkworm exuviae treated by 0.5 U CbPro; blue triangles, silkworm exuviae treated by 0.1 U BbChi3250 + 0.5 U CbPro.

No significant degradation of silkworm exuviae was observed in any group during the first 4 h (Fig. 9). After 8 h of treatment, the total weight of exuviae treated with BbChi3250 (0.1 U) alone decreased by 0.45%, whereas the weight of exuviae treated with CbPro (0.5 U) alone and a combination of BbChi3250 (0.1 U) and CbPro (0.5 U) declined by 9.25% and 8.91%, respectively. By 12 h, the silkworm exuviae treated with BbChi3250 (0.1 U) alone retained 98.85% of their original weight, compared with 86.00% for those treated with CbPro (0.5 U) alone and 76.95% for those treated with a combination of enzymes. After 16 h, silkworm exuviae retained 92.97% of their weight under the BbChi3250 only treatment, 59.71% under CbPro only, and 57.24% under the combined treatment. These results demonstrate that the combination of BbChi3250 and CbPro was particularly efficient in degrading exuviae. Moreover, it is worth mentioning that we have vortexed the silkworm exuviae with Na_2_HPO_4_–citric acid buffer (100 mM, pH 5.0) and measured the chitinase activity in the supernatant, but no detectable activity was observed; the supernatant was further used to co-incubate with BbChi3250, and the activity assay revealed that the BbChi3250 was not inhibited compared with that incubated with buffer (data not shown).

### SEM analysis of enzyme-treated and untreated silkworm exuviae

**Fig 10:**
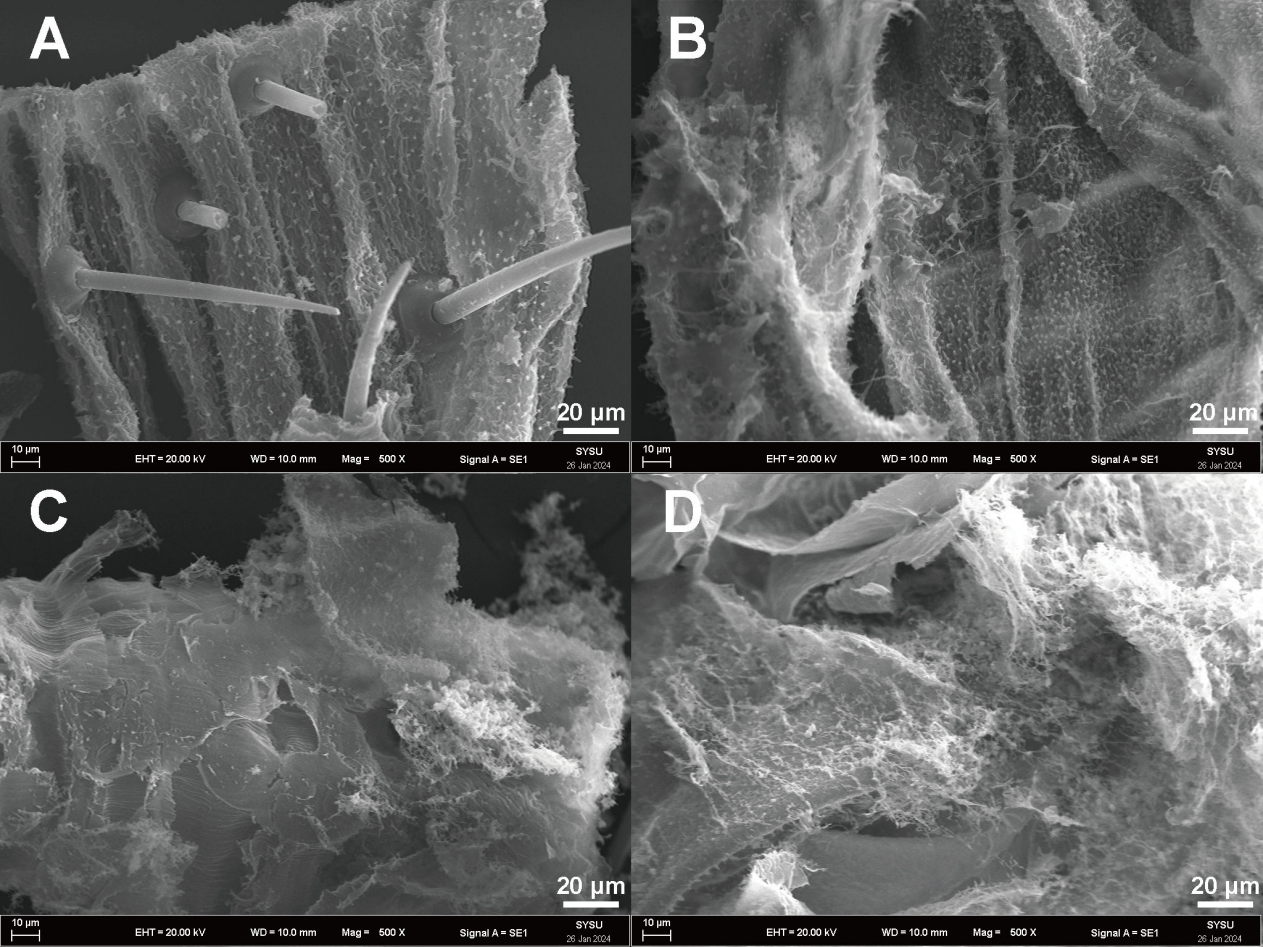
SEM analysis of untreated silkworm exuviae (**A**) and silkworm exuviae treated with 0.1 U BbChi3250 (**B**), 0.5 U CbPro (**C**), and 0.1 U BbChi3250 + 0.5 U CbPro (**D**).

As shown in Fig. 10A, surfaces were intact and compact in untreated silkworm exuviae. In contrast, surface microfibers on BbChi3250-treated silkworm exuviae were depolymerized, generating a lot of microfibrils (Fig. 10B). Likewise, the hydrolysis of silkworm exuviae by CbPro caused cracks and numerous fibrils to form on the surface (Fig. 10C). Furthermore, surfaces appeared fluffier and more irregular under treatment with BbChi3250 + CbPro, indicating more intense depolymerization (Fig. 10D). The SEM images, which are more intuitive and persuasive than values, demonstrated the *in situ* surface ablation and depolymerization activities of BbChi3250 and CbPro on silkworm exuviae, as well as the synergistic effect between the two enzymes. Previously, the investigations of enzyme synergy were generally based on the results of biochemical assays (absorbance data or chromatography analysis data), the innovative combination of biochemical quantitative assays and intuitive SEM analysis in this study has set a precedent for investigating multi-enzyme interactions.

In conclusion, the results demonstrate that BbChi3250 is a new GH18 chitinase that is widely present in *Beauveria* and *Cordyceps* species. This enzyme is an acidic and mesophilic *endo*-chitinase capable of hydrolyzing chitin substrate and silkworm exuviae, displaying a high *k*_cat_/*K*_m_ value of 596.00 mL·mg^–1^·s^–1^ toward EGC. Activity assay and SEM analysis confirmed for the first time that BbChi3250 and CbPro interacted synergistically in the hydrolysis of cuticular chitin and protein in silkworm exuviae. The highest DS values obtained for GlcNAc and tyrosine equivalent yields were 3.41 and 2.55, respectively. This study expands the repertoire of GH18 chitinase, clearly elucidates the role of BbChi3250 in silkworm cuticle degradation, and underscores the importance of synchronous action of chitinase and protease, which together provide a basis for the potential use of this enzyme in the production of *Bombyx batryticatus* by enhancing infection efficacy of *B. bassiana* toward silkworm. Furthermore, the thorough characterization of BbChi3250, including pH/temperature optima, substrate specificity, kinetic parameters, and synergy assay, enhances the reproducibility and utility of the study.

## MATERIALS AND METHODS

### Strain and chemicals

*B. bassiana* KW1 was previously isolated from *Bombyx batryticatus* and stored in our lab. Chitin sourced from crab shells was purchased from Sangon Biotech (Shanghai, China). Ethylene glycol chitin (EGC) was obtained from FUJIFILM Wako Pure Chemical Corporation (Osaka, Japan). Chitooligosaccharides were purchased from Megazyme (Bray, Ireland). Silkworm exuviae were collected from fourth or fifth instar silkworm larvae. Chitin and silkworm exuviae were individually powdered using an electric pulverizer (Hehui Electronic Technol, Xiamen, China), the granules that passed a 40-mesh sieve were collected and designated as powdered chitin and powdered silkworm exuviae, respectively.

Colloidal chitin (CC) was prepared by modifying the protocol described by Chen et al. (15). Briefly, 2 g of powdered chitin was mixed with 80 mL of concentrated hydrochloric acid and mechanically agitated at 4°C for 24 h using a rotary shaker set to 200 revolutions per minute (rpm). Next, 300 mL of ice-cold 50% (vol/vol) ethanol was added to the solution, and the resultant mixture was agitated at 160 rpm at 4°C for 1 h before resting at 4°C for 24 h. The precipitate was collected after centrifuging at 4°C for 5 min at 10,621 × *g* and then rinsed with distilled water until its pH reached 7.0. Finally, the obtained CC was freeze-dried and used to prepare substrate solution (20 mg·mL^–1^). Likewise, acid-treated silkworm exuviae were prepared using the same procedure.

### Gene identification and protein expression

The genome of *B. bassiana* KW1 has been sequenced, and sequence data have been deposited in GenBank under the accession number JBEHWR000000000. After annotating the genome using the Genome Sequence Annotation Server (GenSAS, https://www.gensas.org/) (41), a new gene (*Bbchi3250*) encoding putative chitinase was identified. Subsequently, prediction of conserved domains and a signal peptide was performed using the Conserved Domain Search server (CD-Search, https://www.ncbi.nlm.nih.gov/Structure/cdd/wrpsb.cgi) and SignalP 6.0 server (https://services.healthtech.dtu.dk/services/SignalP-6.0/), respectively (42, 43). Homologous sequences were identified using the BLASTp server (https://blast.ncbi.nlm.nih.gov/Blast.cgi) (44). Phylogenetic analysis was performed by using MEGA 11.0 software (45). Structure-based sequence alignment was carried out using Clustal W software module embedded in MEGA 11.0 and exported by ESPript 3.0 server (https://espript.ibcp.fr/ESPript/ESPript/index.php) (46).

*B. bassiana* KW1 strain was inoculated on Sabouraud dextrose agar plates supplemented with yeast extract (Oxid, Hampshire, UK). Following incubation at 25°C for 14 days at 85% ambient humidity, fresh mycelia were scraped from the plates and quickly frozen by adding liquid nitrogen. The obtained frozen mycelia were immediately ground to a fine powder manually using a mortar and pestle. Subsequently, total RNA was extracted from the mycelia powder using Hipure Plant RNA Mini Kit (Magen Biotech, Guangzhou, China), after which a cDNA library was generated from total RNA using FastKing RT Kit (Tiangen Biotech, Beijing, China). Next, the coding sequence for BbChi3250 was amplified from the cDNA library using PrimeSTAR HS DNA Polymerase (Takara, Dalian, China) and polymerase chain reaction. Primers used were BbChi3250-F-*Nde*I (5′-CGCGCGGCAGCCATATGATGAGTTATCGATCCGTAGCCTATTACG-3′) and BbChi3250-R-*Xho*I (5′-GGTGGTGGTGCTCGAGTAGTTGTCGGGAATCCGTTTCTCA-3′) (*Nde*I and *Xho*I cleavage sites are underlined). Amplicons were digested using *Nde*I/*Xho*I (Takara, Dalian, China) and then ligated into the same digested pET28a(+) vector (Merck, Darmstadt, Germany) using a DNA Ligation Kit Ver. 2.1 (Takara, Dalian, China). Protein expression was performed as described in our previous report (38). Protein purification and concentration determination were performed using TALON® metal affinity resins (Clontech, Mountain View, USA) and BCA protein assay kit (Tiangen, Beijing, China), respectively. Crude cell lysate, supernatant of the cell lysate, and the purified protein were analyzed using sodium dodecyl sulfate-polyacrylamide gel electrophoresis (SDS-PAGE); 5% stacking gel and a 12% separation gel were prepared by following a Cold Spring Harbor Protocol (47). Electrophoresis was performed at a current of 30 mA for 20 min, followed by 68 mA for 45 min. Afterward, proteins were stained with Coomassie brilliant blue G-250 (Biosharp, Hefei, China). All kits were used by following the manufacturer’s protocols.

### Zymography and chitinase activity assay

Zymography was performed using a 5% normal stacking gel and a modified 12% separation gel containing 1 mg·mL^–1^ of EGC by referring to the previously reported with slight modifications (16). Electrophoresis was performed at a current of 20 mA for 3 h at 4°C, after which, the gel was submerged in 100 mL of refolding buffer [0.1 M Na_2_HPO_4_–citric acid (pH 5.0) containing 1% Triton X-100] for 1 h at room temperature to eliminate the SDS and separation buffer in the gel. Subsequently, the gel was incubated in 100 mL 0.1 M Na_2_HPO_4_–citric acid buffer (pH 5.0) at 30°C overnight for enzymatic hydrolysis of substrate. Next, the gel was rinsed with distilled water and stained with Coomassie brilliant blue G-250 (Biosharp, Hefei, China) for 10 min. The gel was then rinsed with distilled water again and placed on the Gel View 1500 Pro (Biolight Biotechnology Co., Ltd., Guangzhou, China) for image collection. A negative control was produced following the same protocol using bovine serum albumin (BSA).

The chitinase activity assay was performed by determining the amount of GlcNAc equivalents (a kind of reducing sugar) released from CC. Reaction mixtures (200 µL) were prepared by mixing 10 µL of 20 µM enzyme solution and 190 µL of 100 mM Na_2_HPO_4_–citric acid buffer (pH 5.5) containing CC at a concentration of 5.26 mg/mL. The mixture was incubated at 30°C for 15 min, after which the reaction was terminated by heating at 100°C for 10 min. The resulting reducing sugars were then quantified using the *p-*hydroxybenzoic acid hydrazide (*p*HBAH) method (48). Briefly, 200 µL of reaction mixture was made by mixing 50 µL of enzymatic hydrolysate and 150 µL of *p*HBAH solution (at a concentration of 1 mg/mL), after which it was heated at 100°C for 10 min. Next, the reaction mixture was mixed with an equal volume of 0.4 M sodium hydroxide-0.1 M sodium citrate buffer, and then, the absorbance was determined spectrophotometrically at the wavelength of 400 nm. A standard curve of GlcNAc was constructed for the calculation of reducing equivalents of GlcNAc. One unit (1 U) of chitinase activity was defined as the amount of enzyme required to liberate 1 µmol of GlcNAc equivalents in one minute. Specific activity is denoted as units per mg of enzyme.

### Effect of pH, temperature, and substrate on the performance of BbChi3250

The optimal pH was determined by measuring chitinase activities as described above, but the buffer was replaced with a series of 100 mM Na_2_HPO_4_–citric acid buffers with different pH (3.0–8.0). Likewise, the optimum reaction temperature was determined by measuring the chitinase activities at temperatures between 10 and 60°C (5°C intervals). Relative activity was obtained by dividing the enzyme activity under each condition by the maximum activity. After determining the optimal pH and temperature, the standard conditions for chitinase activity assay were set as follows: purified BbChi3250 (1 µM) was incubated with substrate (5 mg·mL^–1^) at pH 5.0 and 30°C for 15 min.

To evaluate the pH stability, the purified BbChi3250 was pre-incubated in buffers with different pH [100 mM Na_2_HPO_4_–citric acid buffer (pH 3.0–8.0), 100 mM Tris-HCl buffer (pH 8.0–9.0), 100 mM glycine-NaOH buffer (pH 9.0–11.0)] at room temperature for 12 h. Residual enzyme activity toward CC was assayed under standard conditions, and all activities were reported relative to the activities of untreated enzymes under similar conditions. To evaluate the thermal stability, the purified BbChi3250 was pre-incubated at 30, 35, and 40°C for different periods, after which residual enzyme activity toward CC was assayed under standard conditions. Chitinase activity of untreated BbChi3250 was taken as 100%.

To evaluate the effect of CC or EGC on the enzyme’s thermodynamic stability, the melting temperatures (*T*_m_) for BbChi3250 with or without substrate were measured according to the procedure described in our previous report with minor modifications (49). Briefly, the final concentrations used for enzyme and substrate were changed to 10 µM and 5 mg·mL^–1^, respectively.

### Analyses of substrate specificity, hydrolysates, and kinetic parameters

Various substrates, including CC, EGC, powdered chitin, acid-treated silkworm exuviae, and powdered silkworm exuviae, were used to assess the substrate specificity. Enzyme activities toward different substrates were assayed under standard conditions, with substrates incubated without enzyme used as controls.

To analyze the hydrolysates of chitooligosaccharides [diacetyl-chitobiose (GlcNAc)_2_, triacetyl-chitotriose (GlcNAc)_3_, and tetraacetyl-chitotetraose (GlcNAc)_4_], CC, EGC, and acid-treated silkworm exuviae, each substrate was incubated with the enzyme at pH 5.0 and 30°C, with a shaking speed of 700 rpm. The final concentration of BbChi3250 in each assay was 10 µM, and the final concentrations of chitooligosaccharides and polysaccharide substrates were 1.8 mg·mL^–1^ and 5 mg·mL^–1^, respectively. After 4 h, all reactions were terminated by boiling at 100°C for 10 min. Then, each sample was filtered through a 0.22 µm syringe filter after cooled down and analyzed on a Dionex ICS 5000 + high-performance anion-exchange chromatography configured with a pulsed amperometric detector, a CarboPac PA10 guard column (4 × 50 mm), and a CarboPac PA10 analytical (4 × 250 mm) column (HPAEC-PAD, Thermo Scientific, Sunnyvale, USA). Two mobile phases (mobile phase A, 0.02 M sodium hydroxide solution; mobile phase B, 0.1 M sodium hydroxide–0.5 M sodium acetate solution) were employed for elution. Hydrolysates were eluted as follows: (i) 0–50 min, mobile phase A, (ii) 51–70 min, mobile phase B, and (iii) 71–90 min, mobile phase A. Flow rate and column temperature were set to 0.6 mL·min^–1^ and 25°C, respectively.

Michaelis–Menten kinetic parameters for BbChi3250 were determined using substrate saturation assays. Enzyme solutions with proper concentrations were incubated with various concentrations of CC (0–16 mg·mL^–1^) or EGC (0–1 mg·mL^–1^) under its optimal pH and temperature for 5, 10, and 15 min. The GlcNAc equivalents produced in each assay were then measured, and the initial velocities were calculated and used to construct a scatter plot together with corresponding substrate concentrations using GraphPad Prism software version 8.0 (San Diego, CA, USA). After generating a well-fitted non-linear regression curve using the Michaelis–Menten equation, the half-saturation constant (*K*_m_), turnover number (*k*_cat_), and catalytic efficiency (*k*_cat_/*K*_m_) were derived.

### Prediction of the three-dimensional structure of BbChi3250 and the complex structure of BbChi320 with (GlcNAc)_4_

A three-dimensional model of the structure of BbChi3250 was predicted using the online AlphaFold v2.3.2 server (https://deepmind.google/technologies/alphafold/) (50). To predict the structure of the BbChi3250-(GlcNAc)_4_ complex, the molecular structure of (GlcNAc)_4_ was first retrieved from the structure of a GH19 chitinase from *Bryum coronatum* (BcChi-A) in complex with (GlcNAc)_4_ [Protein Data Bank (PDB) code: 3WH1] (51) and then docked with BbChi3250 using AutoDock 4.2 program (La Jolla, CA, USA), with an optimized binding free energy (52). All protein structural figures were generated using PyMOL v2.5.7 (Schrödinger, New York, USA).

### Synergistic effects of BbChi3250 and a commercial protease on the hydrolysis of silkworm exuviae

To investigate the synergy between chitinase and protease on silkworm exuviae, a commercial protease (designated CbPro) was obtained from Challenge Biotech (Beijing, China) and used in the subsequent assay. CbPro is produced through submerged fermentation of a selected *Bacillus subtilis* strain. This enzyme preparation mainly includes endopeptidase, a small amount of aminopeptidase, and carboxypeptidase. To prepare the enzyme solution, 300 mg of CbPro powder was dissolved in 1.2 mL of 100 mM Na_2_HPO_4_–citric acid buffer (pH 7.0) and thoroughly mixed by vortexing; then, the suspension was centrifuged at 13,523 × *g* at 4°C for 1 min, and the resulting supernatant was collected and used for subsequent assays. The protease activity of CbPro was determined at pH 7.0 and 30°C according to the Folin-Ciocalteu method (53). A standard curve of tyrosine was constructed for the calculation of tyrosine equivalents released by enzymatic hydrolysis. One unit (1 U) of protease activity was defined as the amount of enzyme required to produce 1 µg of tyrosine equivalents per minute. The chitinase activity assay was performed at pH 7.0 and 30°C for 15 min, using acid-treated silkworm exuviae as substrate.

Cooperative hydrolysis of powdered silkworm exuviae by BbChi3250 and CbPro was carried out at 30°C in 100 mM Na_2_HPO_4_–citric acid buffer (pH 7.0). 200 µL of reaction mixture was prepared in a 2 mL Eppendorf tube and then placed in a TCS10 ThermoMixer (Ruicheng, Hangzhou, China) and shaken at 700 rpm. The final concentration of powdered silkworm exuviae was 5 mg·mL^–1^ in all tests. The synergistic effect of CbPro on silkworm exuviae degradation by BbChi3250 was assessed using three different ratios of CbPro and BbChi3250, which were maintained by keeping the enzyme load of BbChi3250 constant (0.1 units) while adding varying amounts of CbPro (0.1, 0.5, or 1 units) in different tests. Meanwhile, reactions using either BbChi3250 (0.1 units) or CbPro (0.1, 0.5, or 1 units) alone were carried out under the same conditions and used as controls; substrate incubated without enzyme was used as a blank. After 4 h of hydrolysis, samples were boiled for 10 min before they were used in the assay of GlcNAc equivalents. Similarly, the synergistic effect of BbChi3250 on the degradation of silkworm exuviae by CbPro was investigated by following the above-described procedure with minor modifications: (i) 0.5 units of CbPro and three different amounts of BbChi3250 (0.05, 0.1, or 0.2 units) were used for the cooperative hydrolysis, and (ii) the reactions were terminated by adding 200 µL trichloroacetic acid and incubating at 40°C for 10 min, after which tyrosine equivalents were quantified using the Folin-Ciocalteu method. The degree of synergy (DS) was calculated using the following formula:


$$\text{Degree of synergy=}\frac{{\text{A}}_{\text{(BbChi3250+CbPro)}}}{{\text{A}}_{\text{BbChi3250}}\text{+}{\text{A}}_{\text{CbPro}}}$$

When the equation is used to evaluate the synergistic effect of CbPro on BbChi3250, A_(BbChi3250+CbPro)_ represents the total amount of GlcNAc equivalents released under the combination of BbChi3250 and CbPro, whereas A_BbChi3250_ and A_CbPro_ denote the amount of GlcNAc equivalents released by either BbChi3250 or CbPro. Similarly, when evaluating the synergistic effect of BbChi3250 on CbPro, A_(BbChi3250+CbPro)_, A_BbChi3250_, and A_CbPro_ denote the amounts of tyrosine equivalents produced by the BbChi3250 + CbPro complex, BbChi3250, and CbPro, respectively. Values of DS greater than 1 indicate synergy between BbChi3250 and CbPro. Conversely, values of DS less than or equal to 1 suggest either no synergy or anti-synergistic interaction between the enzymes.

### Time course analysis of the effects of BbChi3250 combined with a commercial protease on the hydrolyses of silkworm exuviae

To compare the degradation efficiencies of BbChi3250, CbPro, and their combinations on silkworm exuviae over time, reactions were conducted at 30°C in 100 mM Na_2_HPO_4_–citric acid buffer (pH 7.0) for 1, 2, 4, 8, 12, and 16 h; 1 mL of reaction mixture was prepared in a 5 mL Eppendorf tube and shaken in a TCS10 ThermoMixer (Ruicheng, Hangzhou, China) at 700 rpm. A final concentration of 10 mg·mL^–1^ powdered silkworm exuviae was used in all tests; 0.1 units of BbChi3250 and 0.5 units of CbPro were combined for experimental tests. Meanwhile, reactions using BbChi3250 (0.1 units) or CbPro (0.5 units) individually under identical conditions were used as controls, with substrate incubated without enzyme as a blank. After reactions were completed, samples were dried to constant weight and then weighed using an analytical balance (Sartorius, Beijing, China).

### Scanning electron microscopy (SEM) analysis of silkworm exuviae hydrolysis

To visualize and assess the synergistic effects between BbChi3250 and CbPro on the hydrolysis of silkworm exuviae, 0.1 units of BbChi3250 and 0.5 units of CbPro were incubated separately or together with powdered silkworm exuviae (at a final concentration of 5 mg mL^–1^) at pH 7.0 and 30°C in a TCS10 ThermoMixer with a shaking speed of 700 rpm as described above. Meanwhile, the substrate incubated without the enzyme was incubated under the same conditions and used as a blank. After 12 h of incubation, exuviae samples were collected by centrifuging at 18,407 × *g* for 10 min and further freeze-dried overnight. Afterward, the tested samples were prepared for SEM by spreading dried sample particles on a copper grid coated with a carbon support film, after which they were coated with gold. Micrographs showing surface morphology were generated using an EVO MA10 (ZEISS, Oberkochen, Germany) at 20 kV.

### Statistical analyses

All tests were repeated three times, and data were expressed as the mean ± standard deviation. One-way multivariate analysis of variance (ANOVA) was performed using GraphPad Prism software version 8.0 (San Diego, CA, USA), significance of difference between data were assessed at three different *P*-value levels: ***, very significant difference (*P* < 0.001); **, significant difference (0.001 < *P* < .01); *, statistical difference (0.01 < *P* < .05); NS, non-significant difference (*P* > 0.5).

## SUPPLEMENTAL MATERIAL

Supplemental material aem.01285-25-s0001.doc Tables S1 and S2; Fig. S1.

ASM does not own the copyrights to Supplemental Material that may be linked to, or accessed through, an article. The authors have granted ASM a non-exclusive, world-wide license to publish the Supplemental Material files. Please contact the corresponding author directly for reuse.
