# Supplementary material for: Biochemical analyses of a new GH18 chitinase from Beauveria bassiana KW1 and its synergy with a commercial protease on silkworm exuviae hydrolysis
Source: Appl Environ Microbiol. 2025 Sep 15;91(10):e01285-25. doi: 10.1128/aem.01285-25 (PMC12542771; doi:10.1128/aem.01285-25)
Supplement: Supplemental material — Tables S1 and S2; Fig. S1. [file aem.01285-25-s0001.doc]

**Supporting Material**

Biochemical analyses of a new GH18 chitinase from *Beauveria bassiana* KW1 and its synergy with a commercial protease on silkworm exuviae hydrolysis

Yizhou Liu^1,2,†^, Wenke Xiong^1,2,†^, Xinhai Wang^3,†^, Shuangcheng Liang^1,2^, Lixin He^1,2^, Xiaoqin Lin^1,2^, Sidi Wang^4^, Ying Zhang^5^,*, Ruoting Zhan^1,2^,*, Kui Wang^1,2^,*

^1^Research Center of Chinese Herbal Resource Science and Engineering, School of Pharmaceutical Sciences, Guangzhou University of Chinese Medicine, Guangzhou 510006, Guangdong, China. ^2^Key Laboratory of Chinese Medicinal Resource from *Lingnan* (Guangzhou University of Chinese Medicine), Ministry of Education of the People’s Republic of China, Guangzhou 510006, Guangdong, China. ^3^Department of General Surgery, Huashan Hospital of Fudan University, Shanghai 200040, PR China. ^4^College of Fundamental Medical Sciences, Guangzhou University of Chinese Medicine, Guangzhou 510006, Guangdong, China. ^5^School of Pharmaceutical Sciences, Guangzhou University of Chinese Medicine, Guangzhou 510006, Guangdong, China.

^†^ Co–first authors.

* Corresponding authors: Ying Zhang (*Email: tjxyzy@gzucm.edu.cn), Ruoting Zhan (*Email: zhanrt@gzucm.edu.cn), Kui Wang (*Email: kuiwang@gzucm.edu.cn).

**Table S1** Yield of GlcNAc equivalents released from silkworm exuviae by BbChi3250 and CbPro. Assays were replicated three times. Data are presented as mean ± standard deviation.

|  | Group | | | | | | | | |
| --- | --- | --- | --- | --- | --- | --- | --- | --- | --- |
|  | Ⅰ | | | Ⅱ | | | Ⅲ | | |
| BbChi3250 (U) | / | 0.1 | 0.1 | / | 0.1 | 0.1 | / | 0.1 | 0.1 |
| CbPro (U) | 0.1 | / | 0.1 | 0.5 | / | 0.5 | 1.0 | / | 1.0 |
| GlcNAc equivalents (mM) | 0.022 ± 0.019 | 0.039 ± 0.004 | 0.200 ± 0.016 | 0.044 ± 0.008 | 0.039 ± 0.004 | 0.283 ± 0.020 | 0.222 ± 0.008 | 0.039 ± 0.004 | 0.391  ±  0.034 |
| DS | 3.28 ± 1.08 | | | 3.41 ± 0.44 | | | 1.50 ± 0.14 | | |

**Table S2 Yield of tyrosine equivalents released from silkworm exuviae by BbChi3250 and CbPro. Assays were replicated three times. Data are presented as mean ± standard deviation.**

|  | Group | | | | | | | | |
| --- | --- | --- | --- | --- | --- | --- | --- | --- | --- |
|  | Ⅳ | | | Ⅴ | | | Ⅵ | | |
| BbChi3250 (U) | 0.05 | / | 0.05 | 0.1 | / | 0.1 | 0.2 | / | 0.2 |
| CbPro (U) | / | 0.5 | 0.5 | / | 0.5 | 0.5 | / | 0.5 | 0.5 |
| Tyrosine equivalents (mM) | 0.003 ± 0.002 | 0.164 ± 0.010 | 0.253 ± 0.006 | 0.003 ± 0.003 | 0.164 ± 0.010 | 0.293 ± 0.005 | 0.004 ± 0.001 | 0.163 ± 0.010 | 0.425  ±  0.004 |
| DS | 1.52 ± 0.10 | | | 1.75 ± 0.11 | | | 2.55 ± 0.16 | | |

**Figure S1** Kinetic analysis of BbChi3250 hydrolysis on colloidal chitin (A) and ethylene glycol chitin (B).


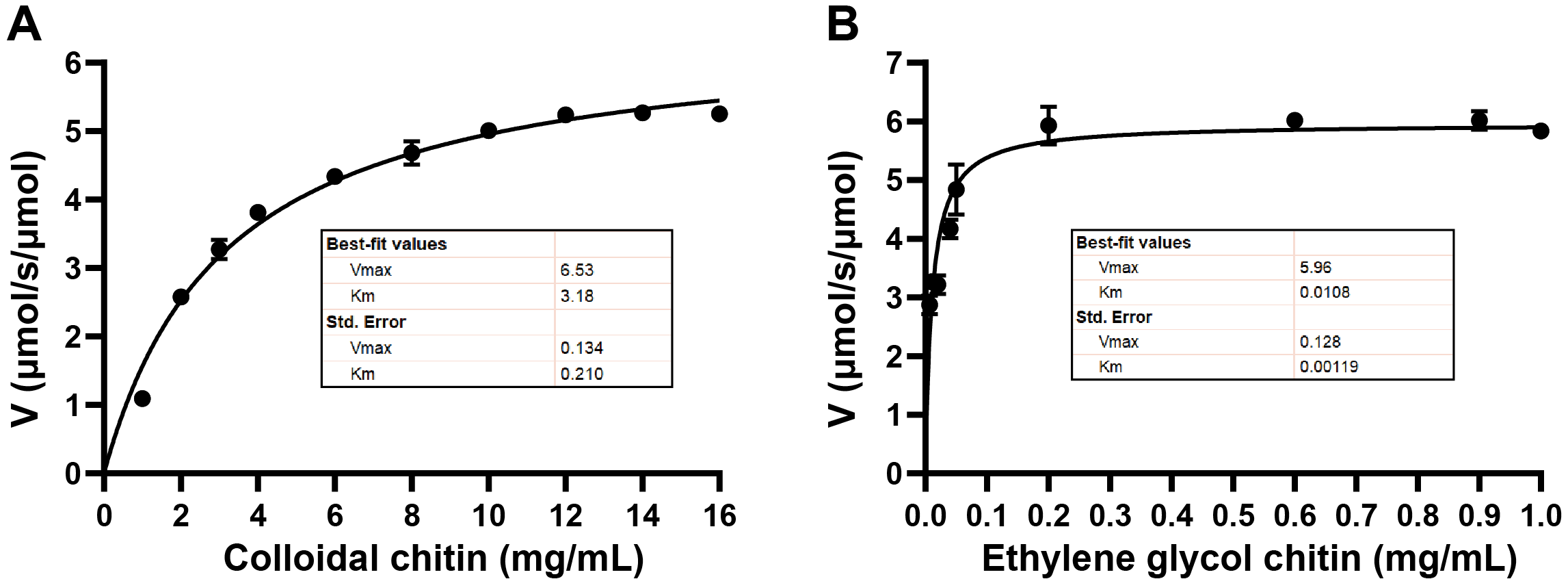


**Figure S1**
